# Anterior cruciate ligament remnant morphology is associated with preoperative rotational knee instability: A multicenter cohort study

**DOI:** 10.1002/jeo2.70650

**Published:** 2026-01-19

**Authors:** Nobuaki Hayashi, Shotaro Watanabe, Tsuyoshi Hamada, Manato Horii, Masahiko Saito, Yuta Muramatsu, Takuya Sakamoto, Yusuke Sato, Taisuke Fukawa, Ryuichiro Akagi, Takuro Moriya, Ryosuke Nakagawa, Seiji Kimura, Satoshi Yamaguchi, Seiji Ohtori, Takahisa Sasho

**Affiliations:** ^1^ Department of Orthopaedic Surgery Graduate School of Medical and Pharmaceutical Sciences Chiba University Chiba Japan; ^2^ Department of Orthopaedic Surgery Center for Preventive Medical Sciences Chiba University Chiba Japan; ^3^ Orthopaedic Surgery, Chiba Medical Center Chiba Japan; ^4^ Orthopaedic Surgery, Kitachiba Spine & Sports Clinic Chiba Japan; ^5^ Orthopaedic Surgery, Eastern Chiba Medical Center Chiba Japan; ^6^ Orthopaedic Surgery, Japanese Red Cross Narita Hospital Chiba Japan; ^7^ Orthopaedic Surgery, Oyumino Central Hospital Chiba Japan; ^8^ Orthopaedic Surgery, Chiba Rosai Hospital Chiba Japan; ^9^ Orthopaedic Surgery, Kohnodai Hospital Chiba Japan; ^10^ Graduate School of Global and Transdisciplinary Studies Chiba University Chiba Japan

**Keywords:** anterior cruciate ligament remnant morphology, Crain classification, Lachman test, pivot‐shift test, rotational knee instability

## Abstract

**Purpose:**

To investigate the association between anterior cruciate ligament (ACL) remnant morphology, classified by Crain, and preoperative rotational knee instability after adjusting for preoperative and anatomical factors including time from injury to surgery and meniscal status. It was hypothesised that remnant morphology would be associated with rotational knee instability.

**Methods:**

This retrospective multicenter cohort study was conducted between October 2022 and June 2025. Arthroscopic assessment categorised ACL remnants according to the Crain classification. Under anaesthesia, the Lachman and pivot‐shift tests were graded and dichotomised as high‐grade or low‐grade. Univariate comparisons were performed using the chi‐square test. Multivariate logistic regression identified predictors of high‐grade Lachman (L‐HG) and pivot‐shift (PS‐HG), adjusting for age, sex, body mass index, time from injury to surgery, mechanism of injury, Tegner Activity Scale (TAS) score, and medial and lateral meniscal tears, with Crain Type 4 as the reference.

**Results:**

Among 304 patients, Crain types were as follows: Type 1 (16.8%), Type 2 (40.8%), Type 3 (27.6%) and Type 4 (14.8%). Overall, L‐HG and PS‐HG occurred in 36.8% and 39.8% of patients, respectively. L‐HG did not differ among the Crain groups (*p* = 0.081), whereas PS‐HG did (*p* = 0.016). In multivariate analysis, TAS score (odds ratio [OR] per unit, 0.83; 95% confidence interval [CI], 0.70–0.98; *p* = 0.028) and lateral meniscal tear (OR, 2.16; 95% CI, 1.31–3.58; *p* = 0.003) were significant predictors of L‐HG. For PS‐HG, Crain Type 3 had lower odds compared with Type 4 (OR, 0.43; 95% CI, 0.19–0.99; *p* = 0.048).

**Conclusions:**

Crain remnant morphology was associated with rotational instability, with Type 3 demonstrating lower odds of high‐grade pivot shift than Type 4. These findings suggest that ACL remnant morphology may play a role in rotational instability.

**Level of Evidence:**

Level IV, cohort study.

## Graphical abstract

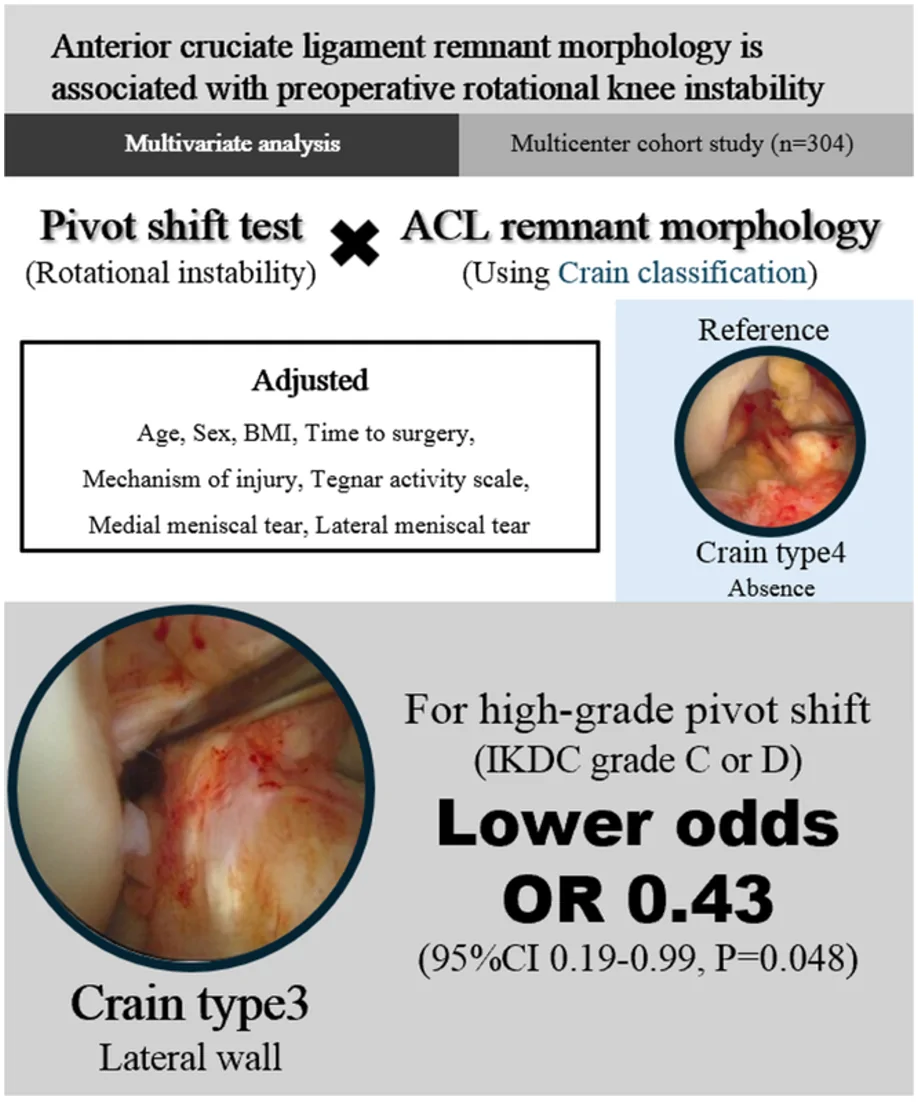

**Abbreviations**
ACL: anterior cruciate ligament
BMI: body mass index
CI: confidence interval
L‐HG: Lachman test high‐grade
OR: odds ratio
PCL: posterior cruciate ligament
PS‐HG: pivot shift test high‐grade
TAS: Tegner activity scale

## INTRODUCTION

The anterior cruciate ligament (ACL) contributes to both anteroposterior and rotational stability of the knee joint. ACL tears occur throughout the ligament, with midsubstance tears most common, proximal tears also frequent, and distal tears uncommon [15]. Based on the proximal reattachment site and amount of preserved tissue, ACL remnants have been categorised into distinct morphological types. Although several classification schemes for ACL remnant morphology have been reported [5, 9, 27], the Crain classification is the most commonly used [5].

The relationship between ACL remnant morphology and anteroposterior and rotational knee instability has been investigated. Crain Type 4 remnants have been reported to show greater anteroposterior instability compared with Type 3, whereas the association between rotational instability and ACL remnant morphology remains inconsistent [5, 16, 20, 21, 22, 25, 26, 28].

In addition to remnant morphology, several preoperative factors have been implicated in knee instability. Younger age has been associated with greater anteroposterior and rotational instability [6, 17], and female sex has been reported to predispose patients to rotational instability [17]. A prolonged interval from injury to surgery has also been correlated with increased anteroposterior and rotational instability [6, 7, 17]. Furthermore, concomitant meniscal injuries have been identified as important contributors to anteroposterior and rotational instability [6, 7, 17]. In light of these findings, a comprehensive multivariate analysis incorporating both ACL remnant morphology and other risk factors is warranted in the assessment of knee instability.

Therefore, this study aimed to investigate the association between ACL remnant morphology and rotational knee instability, adjusting for relevant factors including time from injury to surgery and meniscal status. The hypothesis was that ACL remnant morphology would be associated with rotational instability.

## MATERIALS AND METHODS

### Study design

Prospectively collected data from a multicenter ACL reconstruction study were analysed retrospectively. The protocol was approved by the coordinating institutional review board for all participating centres, and written informed consent for research use of de‐identified study data was obtained from all participants at the time of enrolment.

### Patient selection

Patients who underwent ACL reconstruction at nine participating institutions between October 2022 and June 2025 were included. Inclusion criteria were identical across sites and required complete registry data entry for all eligible ACL reconstruction cases at each institution. All patients underwent manual physical examination and magnetic resonance imaging prior to surgery, and ACL rupture was confirmed arthroscopically. Clinical and imaging evaluations were performed by board‐certified orthopaedic surgeons specialising in knee surgery, each with more than 10 years of clinical experience.

Exclusion criteria were as follows: associated fractures; age ≤ 14 years or ≥ 60 years; revision ACL reconstruction; previous surgery on the affected or contralateral knee; combined ligament injuries (posterior cruciate ligament [PCL] and/or Grade 3 collateral ligament injuries); and radiographic evidence of osteoarthritic changes (Kellgren–Lawrence Grade ≥ 2). Primary ACL reconstructions were included. Laterality was recorded at the index surgery, and bilateral injuries without prior knee surgery were not excluded when each knee independently met eligibility criteria. Patients with Grade 1 or 2 collateral ligament injuries were not excluded. For patients with Grade 1 or 2 collateral ligament injuries, ACL reconstruction was performed after clinical improvement, including resolution of pain and adequate restoration of knee range of motion, to allow recovery of the collateral ligament prior to surgery.

### Data collection and variables

All participating institutions used a standardised registration form to record demographic data, including age, sex, and body mass index (BMI); Tegner Activity Scale (TAS) score; comorbidities; surgical history; cause and date of injury; Lachman test grade; pivot‐shift test grade; date of surgery; ACL remnant morphology; and meniscal injury. Contact injuries were defined as those involving either direct contact with the knee or indirect contact through other body parts contributing to the injury mechanism. Non‐contact injuries were defined as those occurring without any external contact at the time of injury. Registration in the database was considered complete once all required preoperative and intraoperative data were collected. Data entries were shared across participating centres and verified by site investigators to ensure completeness and internal consistency before database lock.

### Evaluation of knee instability

Knee instability under general or spinal anaesthesia was assessed by the surgeon, who also graded the ACL remnant morphology. Lachman and pivot‐shift tests were performed bilaterally to allow side‐to‐side comparison, and grades for the injured knee were assigned according to the IKDC classification [10].

The Lachman test was performed with the knee in 25° of flexion, placing one hand medially on the tibia and the other laterally on the femur. Test results were classified into four grades: Grade A, normal anterior translation, defined as a side‐to‐side difference of −1 to 2 mm; Grade B, nearly normal translation, with a difference of 3–5 mm; Grade C, abnormal translation, with a difference of 6–10 mm; and Grade D, severely abnormal translation, with a difference of > 10 mm [10]. In this study, Grades A and B were considered low‐grade instability, whereas Grades C and D were considered high‐grade instability.

The pivot‐shift test was performed by holding the heel, applying internal rotation and valgus stress to the tibia, and flexing the knee from full extension. Results were graded into four categories: Grade A, a negative pivot shift corresponding to a normal knee; Grade B, a nearly normal pivot shift with a glide (+); Grade C, an abnormal pivot shift with a clunk (++); and Grade D, a severely abnormal pivot shift characterised by a gross shift (+++) [10]. As with the Lachman test, Grades A and B were categorised as low‐grade instability, and Grades C and D as high‐grade instability.

Across institutions, grading criteria and operational definitions for the Lachman and pivot‑shift tests were aligned through inter‑institutional consensus meetings to promote consistency of performance and interpretation.

### Evaluation of intra‐articular injuries

ACL reconstruction was performed under general or spinal anaesthesia. Intra‐articular assessment was conducted through the anterolateral and anteromedial portals using a probe. ACL tears were confirmed arthroscopically. The medial and lateral menisci were probed to evaluate tear size and type, and the findings were recorded. Meniscal injuries requiring repair or resection were classified as meniscal tears.

### Classification of ACL remnants

**Figure 1:**
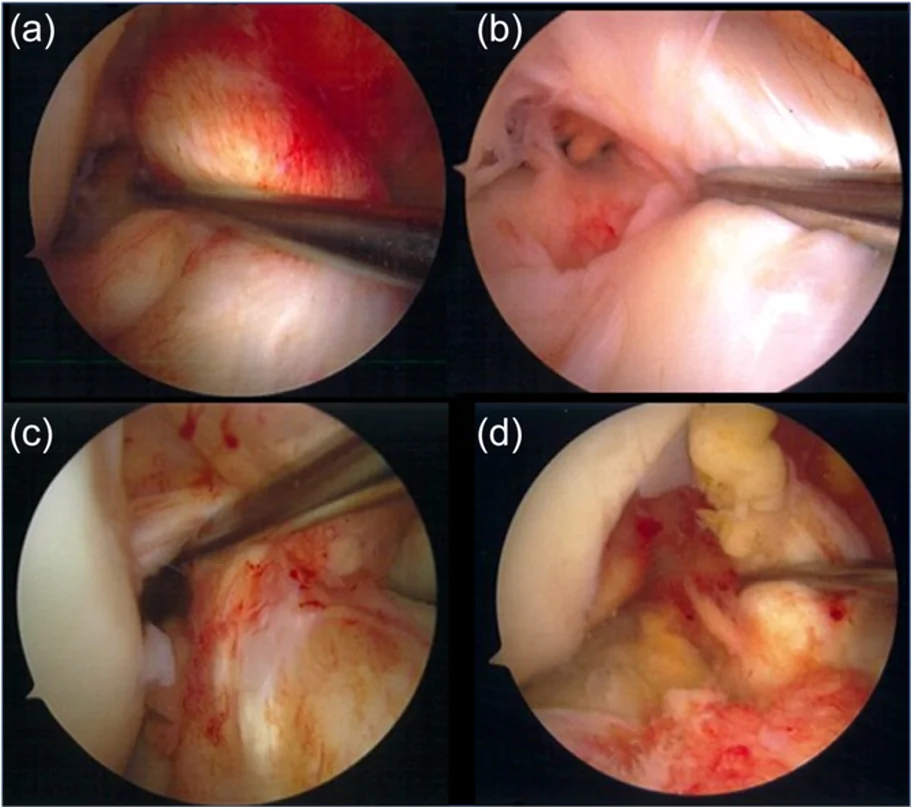
Arthroscopic findings of anterior cruciate ligament (ACL) remnants. (a) Type 1: bridging between the posterior cruciate ligament and the tibia. (b) Type 2: bridging between the roof of the intercondylar notch and the tibia. (c) Type 3: bridging between the lateral wall of the intercondylar notch and the tibia. (d) Type 4: absence of substantial ACL remnants.

ACL remnant morphology was assessed arthroscopically using a probe. According to Crain's classification [5], remnants were categorised into four types based on their morphological patterns. Type 1 was defined as bridging between the PCL and tibia. Type 2 was defined as bridging between the roof of the intercondylar notch and the tibia. Type 3 was defined as bridging between the lateral wall of the intercondylar notch and the tibia. Type 4 was defined as the absence of substantial ACL remnants (Figure 1).

### Statistical analysis

Demographic characteristics (age, sex, BMI, time from injury to surgery, mechanism of injury, TAS and contralateral knee hyperextension) and clinical features (medial and lateral meniscal tears) were summarised by Crain classification. Continuous variables are presented as mean ± standard deviation or median with interquartile range, as appropriate. Categorical variables are presented as numbers (percentages). Continuous variables were analysed using one‐way analysis of variance or the Kruskal–Wallis test, depending on data normality. Categorical variables were analysed using the chi‐square test.

For the primary analysis, univariate and multivariate analyses were conducted for each Crain type to compare the proportions of high‐grade Lachman (L‐HG) and high‐grade pivot‐shift (PS‐HG) groups. Univariate analysis was performed to compare the four groups using the chi‐square test. In multivariate analysis, L‐HG and PS‐HG were set as dependent variables, whereas age, sex, BMI, time to surgery, mechanism of injury, TAS, Crain classification (Type 4 as reference), medial meniscal tear and lateral meniscal tear were included as independent variables. Logistic regression analysis was performed to identify predictors. In multivariate models, Type 4 served as the reference category because it is considered to represent the highest degree of instability, providing a clinically meaningful baseline for comparisons.

Statistical analyses were conducted using JMP®, Version 18 (SAS Institute Inc., Cary, NC, 1989–2025), with statistical significance set at *p* < 0.05.

## RESULTS

### Study participants and group allocation

**Figure 2:**
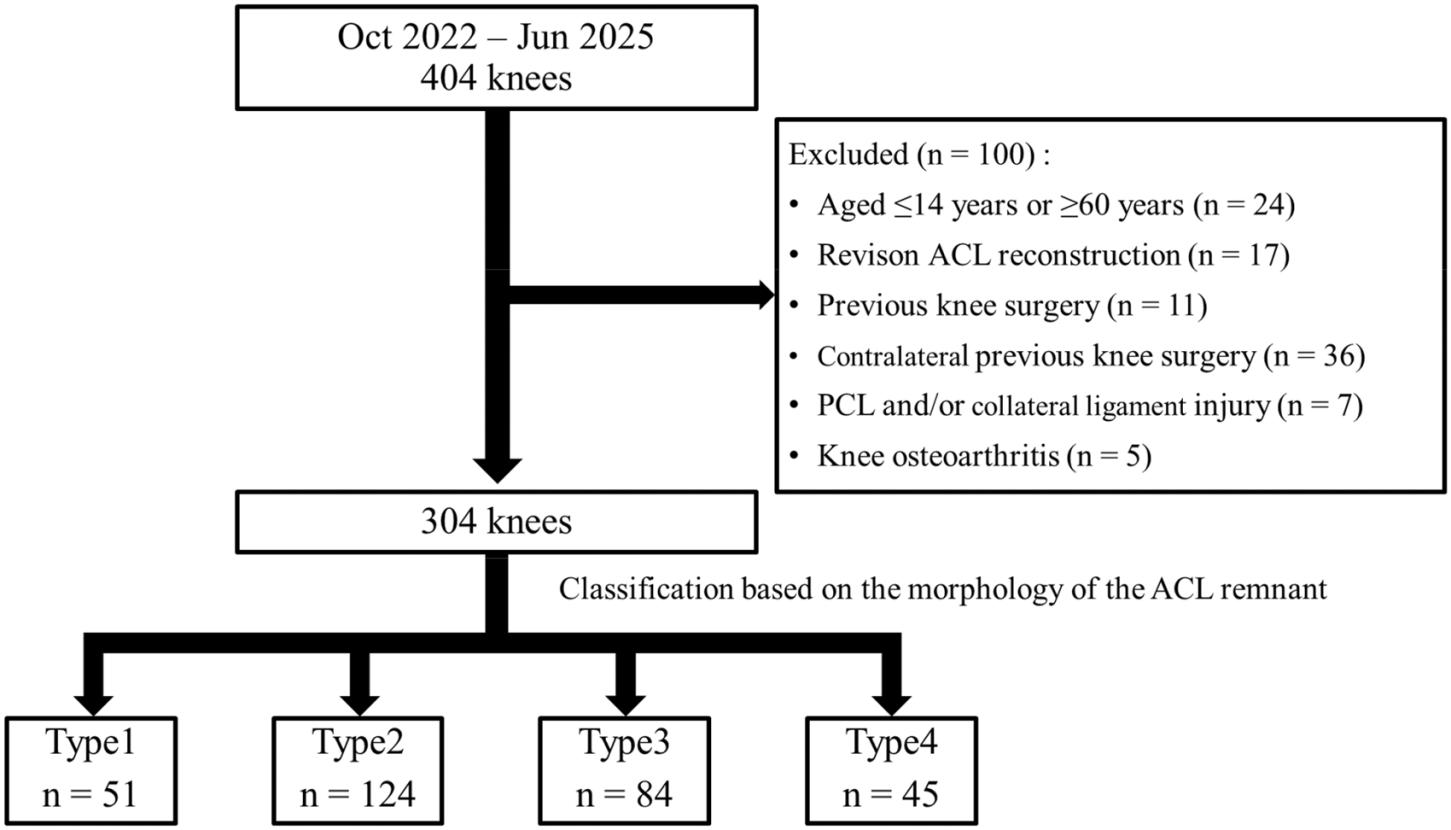
Flowchart of study participants. After exclusion of ineligible patients from the initial 404 cases, the remaining patients were classified into four groups according to the remnant ACL tissue. ACL, anterior cruciate ligament; PCL, posterior cruciate ligament.

A total of 404 patients underwent ACL reconstruction, of whom 100 were excluded based on prespecified exclusion criteria, leaving 304 patients for the final statistical analysis. According to Crain's classification, 51 patients (16.8%) were categorised as Type 1, 124 (40.8%) as Type 2, 84 (27.6%) as Type 3 and 45 (14.8%) as Type 4 (Figure 2).

### Comparison of patient characteristics

Patient characteristics are presented in Table 1. No significant differences were observed among the four Crain groups in terms of age, sex, BMI, mechanism of injury, TAS or lateral meniscal tear. In contrast, significant differences were observed in time to surgery (*p* = 0.038) and medial meniscal tear (*p* < 0.001) between the groups.

**Table 1:** Patient characteristics for all cases and by Crain classification.^a^

	All cases (*n* = 304)	Type 1 (*n* = 51)	Type 2 (*n* = 124)	Type 3 (*n* = 84)	Type 4 (*n* = 45)	*p* value
Age, years	28.6 ± 12.5	29.3 ± 13.6	28.1 ± 12.3	29.8 ± 11.8	27.1 ± 13.0	0.65
Sex						0.87
Male	169 (55.6%)	28 (54.9%)	66 (53.2%)	48 (57.1%)	27 (60.0%)	
Female	135 (44.4%)	23 (45.1%)	58 (46.8%)	36 (42.9%)	18 (40.0%)	
BMI, kg/m^2^	24.0 ± 4.0	23.3 ± 3.3	24.1 ± 4.0	24.2 ± 4.0	24.3 ± 4.5	0.57
Time to surgery, months	2 (1–6)	3 (2–8)	2 (1–5)	2 (1–3)	4 (1–15)	**0.038**
Mechanism of injury						0.45
Contact	79 (26.0%)	13 (25.5%)	38 (30.6%)	18 (21.4%)	10 (22.2%)	
Non‐contact	225 (74.0%)	38 (74.5%)	86 (69.4%)	66 (78.6%)	35 (77.8%)	
Tegner Activity Scale	6 (5–7)	6 (4–7)	7 (6–7)	6 (5–7)	6 (5–7)	0.084
Medial meniscal tear	111 (36.5%)	22 (43.1%)	44 (35.5%)	19 (22.6%)	26 (57.8%)	**<0.001**
Lateral meniscal tear	158 (52.0%)	21 (41.2%)	67 (54.0%)	44 (52.4%)	26 (57.8%)	0.36

Abbreviation: BMI, body mass index.

^a^ Data are presented as mean ± standard deviation, numbers (*n*) with percentage (%), or median with interquartile range. **Bold *p*‐values** indicate statistically significant differences (*p* < 0.05).

### Primary outcomes

#### Incidence of high‐grade knee instability

Overall, L‐HG was detected in 112 (36.8%) patients, whereas PS‐HG was detected in 121 (39.8%). By Crain classification, the incidence of L‐HG was 31.4% in Type 1, 36.3% in Type 2, 32.1% in Type 3 and 53.3% in Type 4, with no significant difference between the groups (p = 0.081). In contrast, the incidence of PS‐HG was 47.1% in Type 1, 38.7% in Type 2, 28.6% in Type 3 and 55.6% in Type 4, showing significant differences between the groups (*p* = 0.016) (Table 2).

**Table 2:** Knee instability for all cases and by Crain classification.^a^

	All cases (*n* = 304)	Type 1 (*n* = 51)	Type 2 (*n* = 124)	Type 3 (*n* = 84)	Type 4 (*n* = 45)	*p* value
Lachman test						0.081
Low‐grade (A or B)	192 (63.2%)	35 (68.6%)	79 (63.7%)	57 (67.9%)	21 (46.7%)	
High‐grade (C or D)	112 (36.8%)	16 (31.4%)	45 (36.3%)	27 (32.1%)	24 (53.3%)	
Pivot‐shift test						**0.016**
Low‐grade (A or B)	183 (60.2%)	27 (52.9%)	76 (61.3%)	60 (71.4%)	20 (44.4%)	
High‐grade (C or D)	121 (39.8%)	24 (47.1%)	48 (38.7%)	24 (28.6%)	25 (55.6%)	

^a^ Data are presented as numbers (n) and ratios (%). **Bold *p*‐values** indicate statistically significant differences (*p* < 0.05).

#### Multivariate logistic regression analysis for knee instability

Patients with lateral meniscal tear had more than twice the odds of L‐HG (odds ratio [OR], 2.16; 95% confidence interval [CI], 1.31–3.58; *p* = 0.003), whereas a higher activity level (TAS) was associated with lower odds (OR per unit, 0.83; 95% CI, 0.70–0.98; *p* = 0.028). The Crain classification was not a significant predictor of L‐HG. For PS‐HG, Crain Type 3 had approximately half the odds compared with Type 4 (OR, 0.43; 95% CI, 0.19–0.99; *p* = 0.048). Crain Types 1 and 2 were not significant predictors compared with Type 4 (Table 3).

**Table 3:** Multivariate logistic regression analysis for knee instability.^a^

	Lachman high‐grade		Pivot‐shift high‐grade	
	OR (95% CI)	*p*‐Value	OR (95% CI)	*p* value
Age	0.98 (0.96–1.00)	0.15	1.00 (0.98–1.02)	0.80
Sex				
Male	1.00 (Reference)		1.00 (Reference)	
Female	0.85 (0.50–1.44)	0.54	1.53 (0.90–2.60)	0.12
BMI	0.98 (0.92–1.04)	0.53	1.03 (0.96–1.10)	0.39
Time to surgery	1.00 (1.00–1.01)	0.39	1.01 (1.00–1.00)	0.052
Mechanism of injury				
Contact	1.00 (Reference)		1.00 (Reference)	
Non‐contact	1.09 (0.60–1.97)	0.79	1.34 (0.73–2.45)	0.35
Tegner Activity Scale	0.83 (0.70–0.98)	**0.028**	1.13 (0.95–1.34)	0.18
Medial meniscal tear	1.51 (0.89–2.54)	0.12	1.40 (0.83–2.35)	0.21
Lateral meniscal tear	2.16 (1.31–3.58)	**0.003**	1.48 (0.90–2.44)	0.12
Crain classification				
Type 4	1.00 (Reference)		1.00 (Reference)	
Type 1	0.46 (0.19–1.11)	0.08	1.00 (0.42–2.36)	1.00
Type 2	0.62 (0.29–1.29)	0.20	0.65 (0.31–1.37)	0.26
Type 3	0.53 (0.24–1.18)	0.12	0.43 (0.19–0.99)	**0.048**

Abbreviations: BMI, body mass index; CI, confidence interval; OR, odds ratio.

^a^ Odds ratios were adjusted for all other predictors. **Bold *p*‐values** indicate statistically significant differences (*p* < 0.05).

## DISCUSSION

In the present study, univariate analysis revealed no significant differences in L‐HG among the four groups, whereas significant differences were observed in PS‐HG. Logistic regression analysis demonstrated that TAS and lateral meniscal tears were significant predictors of L‐HG, whereas Crain classification was not an independent predictor. In contrast, the only significant predictor of PS‐HG was Crain Type 3 compared with Type 4.

Several studies have reported univariate analyses of the relationship between Crain classification and preoperative anteroposterior knee instability [20, 21, 25, 28]. In a study of 100 patients, preoperative anteroposterior instability at 30° and 60° of knee flexion was measured using the KT‐2000 knee ligament arthrometer (MedMetric, San Diego, CA) and OrthoPilot ACL navigation system (Braun Aesculap, Tuttlingen, Germany). Type 3 remnants demonstrated significantly lower instability than Types 1, 2, and 4 when assessed with the KT‐2000 at 30° of knee flexion, whereas no significant differences were observed using the OrthoPilot system [21]. In another study of 121 cases, preoperative anteroposterior instability was assessed during the Lachman test using the KT‐1000 knee ligament arthrometer (MedMetric, San Diego, CA) and a three‐dimensional electromagnetic measurement system (EMS; Polhemus, Colchester, VT) [12]. Type 3 remnants showed significantly smaller instability than Types 2 and 4 using the KT‐1000, and significantly smaller instability than Types 1, 2, and 4 using the EMS [20]. In studies using the Telos stress device (Laubscher, Holstein, Switzerland) [4] at 20° of knee flexion, Type 4 remnants demonstrated significantly greater instability than Types 2 and 3 in one cohort of 123 cases [23], and greater instability than Type 3 in another cohort of 192 cases [25]. In the present study, anteroposterior instability was assessed using the Lachman test, and no significant differences were observed among the groups in univariate analysis. Furthermore, logistic regression analysis demonstrated that Crain classification was not a significant predictor of L‐HG. In contrast, multivariable analysis identified TAS and lateral meniscal tear as the only independent predictors of L‐HG. The inverse association between TAS and L‐HG may reflect the condition of secondary stabilisers such as the anterolateral complex. The positive association between lateral meniscal tear and L‐HG is consistent with prior reports that have shown this relationship [17]. A potential explanation for these findings is that anteroposterior instability was evaluated qualitatively rather than quantitatively in this study. Previous studies have shown that while examiners can reliably distinguish between a firm endpoint and a markedly soft or absent endpoint, their ability to discriminate intermediate grades or quantify anterior tibial translation in millimetres demonstrates only moderate inter‐rater reliability, making subtle differences in instability difficult to detect [18]. This limitation may have contributed to the lack of a significant association between Crain classification and anteroposterior instability observed in the present study.

Several studies have reported univariate analyses of the relationship between Crain classification and preoperative rotational knee instability [5, 20, 21, 28]. In a prospective study of 48 cases, no significant differences in pivot‐shift grade were observed among the groups [5]. In a study of 100 cases, preoperative rotational instability was measured at 30° and 60° of knee flexion using the OrthoPilot system, and no significant differences were found among the groups [21]. Similarly, in 121 patients assessed with EMS during the pivot‐shift test, no significant differences were reported [20]. In contrast, in 192 cases evaluated using pivot‐shift grading, Type 4 remnants demonstrated significantly higher grades than Types 1, 2 and 3 [28]. In the present study, univariate analysis revealed significant differences in rotational instability among the four groups. Furthermore, logistic regression analysis identified Crain Type 3 as a significant predictor of PS‐HG compared with Type 4. These results differ from those of several previous reports, which found no association between the Crain classification and rotational instability. Possible explanations for this discrepancy include differences in evaluation methods and sample sizes across studies. An additional strength of the present study is that the sufficiently large sample size allowed multivariate analyses using PS‐HG as the outcome variable. In multivariable models that adjusted for factors linked to both instability and remnant status, specifically time from injury to surgery and meniscal injury [5, 8, 25, 28], as well as sex and age as correlates of rotational knee instability [6, 17], the Crain classification remained an independent predictor of rotational instability.

The differences in knee stability across Crain types in this study can be interpreted primarily through mechanical considerations. Remnant configurations that preserve greater anatomic continuity are expected to attenuate anterolateral subluxation and reduce rotational instability [29]. Type 4, in which ACL is absent, would be anticipated to provide the least restraint. Only Types 2 and 3 maintain a connection between the femur and the tibia. Type 2, with a more vertical fibre orientation, offers limited rotational constraint and only modest resistance to anterior translation [14]. Type 3 preserves a more anatomical relationship to the femur and may contribute to rotational restraint. Because its femoral attachment tends to be anterior to the native footprint, any contribution to anterior restraint is likely limited. These mechanical considerations are consistent with the finding in the present study that remnant morphology was associated with rotational instability but not with anteroposterior instability. Regarding proprioception, prior studies have reported that mechanoreceptors within the ACL consist predominantly of Type 1 and Type 2 end organs, with a smaller proportion of Type 4 receptors [1, 22]. A reduction in viable mechanoreceptors, particularly in Type 4 remnants with minimal continuity, may weaken dynamic control and increase loading on secondary stabilisers such as the medial meniscus [1, 23].

The clinical implication of these findings is that ACL remnant morphology, particularly Crain Type 3 compared with Type 4, may serve as a predictor of preoperative rotational instability. Recognition of these morphological patterns can assist surgeons in preoperative risk assessment and guide decisions regarding graft selection and augmentation strategies, including reconstruction techniques that preserve remnant tissue. Ultimately, understanding the relationship between remnant morphology and knee instability may help optimise surgical outcomes and reduce the risk of graft failure and reinjury.

## LIMITATIONS

This study has several limitations. First, instability was assessed solely with qualitative clinical tests, which are inherently subjective and may vary among examiners. Because quantitative tools such as arthrometers or navigation systems were not used, subtle differences may have been overlooked [3, 11, 13, 18, 19]. To minimise inter‐observer variability, testing procedures and grading criteria were standardised across participating institutions, and consistency in evaluation was confirmed. Second, the grading of the Lachman and pivot‐shift tests was dichotomised into A or B versus C or D. Although this categorisation was based on previous literature, the chosen cutoff may have influenced the results. Third, although the Crain classification is simple and widely used, its interobserver reliability is not particularly high [26]; furthermore, alternative schemes with greater granularity have been described, which may categorise remnants differently and thus affect cross‑study comparisons [27]. Consistent with previous reports, Crain Type 2 was the most common remnant type in the present study [2, 25, 28]. Fourth, potential confounding by time from injury to surgery should be acknowledged. Although this variable was adjusted for in multivariable analyses, the wide range may permit evolution of remnant morphology [24]. Fifth, the retrospective design introduces the possibility of selection bias and unmeasured confounders. In particular, the current study included only patients aged 15–59 years who underwent primary ACL reconstruction without advanced osteoarthritis, major concomitant injuries, or prior knee surgery; therefore, nonoperatively treated patients and older, lower‐demand individuals with chronic ACL deficiency were not represented. Finally, the absence of postoperative outcomes further limits conclusions regarding long‐term prognostic implications. Although the overall sample size was relatively large, the subgroup analyses may still have lacked sufficient power to detect smaller effects.

## CONCLUSION

In this study, Crain remnant morphology was associated with rotational instability, with Type 3 demonstrating lower odds of high‐grade pivot shift than Type 4. These findings suggest that ACL remnant morphology may play a role in rotational instability.

## AUTHOR CONTRIBUTIONS

Conception and design: Nobuaki Hayashi, Shotaro Watanabe, and Takahisa Sasho. Analysis and interpretation of data: Nobuaki Hayashi, Shotaro Watanabe, and Takuya Sakamoto. Acquisition of data: All authors. Drafting of the article: Nobuaki Hayashi and Shotaro Watanabe. Critical revision of the article for important intellectual content and final approval: All authors. Accuracy of the work: All authors. Funding acquisition: Nobuaki Hayashi and Shotaro Watanabe. All authors had full access to all data in the study and take responsibility for the integrity of the data and the accuracy of the data analysis.

## CONFLICT OF INTEREST STATEMENT

Seiji Ohtori has received lecture fees and honoraria from Daiichi Sankyo Co., Ltd., Nippon Zoki Pharmaceutical Co., Ltd., and Hisamitsu Pharmaceutical Co., Inc.

## ETHICS STATEMENT

The study protocol was approved by the Ethics Committee of Chiba University Hospital (Approval No. M10346) and the ethics committees of all participating institutions. Written informed consent was obtained from all patients prior to surgery.

## Data Availability

Data supporting the findings of this study are available from the corresponding author upon reasonable request.
